# Efficacy and Tolerability of First Line Arsenic Trioxide in Combination With All-Trans Retinoic Acid in Patients With Acute Promyelocytic Leukemia: Real Life Experience

**DOI:** 10.3389/fonc.2021.614721

**Published:** 2021-07-16

**Authors:** Francesco Autore, Patrizia Chiusolo, Federica Sorà, Sabrina Giammarco, Luca Laurenti, Idanna Innocenti, Elisabetta Metafuni, Nicola Piccirillo, Livio Pagano, Simona Sica

**Affiliations:** ^1^ Dipartimento di Diagnostica per Immagini, Radioterapia Oncologica ed Ematologia, Fondazione Policlinico Universitario A. Gemelli IRCCS, Roma, Italy; ^2^ Sezione di Ematologia, Dipartimento di Scienze Radiologiche ed Ematologiche, Università Cattolica del Sacro Cuore, Roma, Italy

**Keywords:** acute promyelocytic leukemia, arsenic trioxide, all-trans retinoic acid, first-line, secondary leukemias

## Abstract

Acute promyelocytic leukemia is a variant of acute myeloid leukemia characterized by t(15;17) and PML/RAR alfa fusion gene. The discovery of the molecular pathogenesis has led to entitle all-trans retinoic acid (ATRA) as the first targeted therapy for acute leukemia. It is usually associated to anthracycline-based chemotherapy with high response rates, but potential long-term sequelae including therapy-related malignancies have been observed. Arsenic trioxide (ATO) was added to obviate these complications and investigational trials aimed to a new strategy with the incorporation of arsenic trioxide (ATO) into initial therapy instead of chemotherapy in selected patients. ATRA plus ATO without chemotherapy was the first attempt to treat low and intermediate-risk patients with APL. Our study aims to describe a monocentric cohort of patients with newly diagnosed APL effectively treated with ATO plus ATRA underlying its efficacy together with the high grade of tolerability of this association. From January 2009 to December 2019 23 APL patients were diagnosed and treated with ATO plus ATRA regimen: 14 males and 9 females patients with a median age of 45 years (range 18-72), for the majority intermediate risk (15 patients, 65%). The treatment was well tolerated and all patients achieved molecular remission after a median time of 3 months (range 1-6 months). All patients proceeded to consolidation phase as outpatients, they maintained complete molecular response at a median time of 44 months (range 15-127) except for 1 patient. All but one patient are alive and in response at a median follow-up of 48 months (range 9-141) without late effects. ATO plus ATRA regimen shows advantages in comparison to chemotherapy; in fact it allowed to treat patients in which chemotherapy could even not be applicable and it did not show secondary hematological diseases. The association of ATO to ATRA as chemo-free regimen enabled to treat APL even without chemotherapy.

## Introduction

In the setting of acute myeloid leukemia (AML) acute promyelocytic leukemia (APL) is a variant characterized by t(15;17) and PML/RAR alfa. All-trans retinoic acid (ATRA) was used as a targeted therapy on the basis of this molecular transcript and was usually associated to anthracycline-based chemotherapy (1). This treatment achieved overall remission rates of up to 95% and cure rates over 80% (2–4). Italian GIMEMA and Spanish PETHEMA trials demonstrated a high antileukemic efficacy of this protocol in terms of complete remission (CR) and disease-free survival rates (1). All these patients received induction according to AIDA schedule (5), 3 courses of consolidation and then maintenance. For consolidation, GIMEMA patients received 3 courses with idarubicin/cytarabine, mitoxantrone/etoposide, and idarubicin/cytarabine/thioguanine respectively, whereas PETHEMA patients received the same drugs and dose schedule of idarubicin and mitoxantrone without non-intercalating agents (3, 6). Data of the Spanish group indicated that, to reduce significantly toxicity, might be used in APL a less intensive consolidation and this seemed not to compromise the antileukemic effect. A minor role for cytarabine and etoposide was also suggested in the treatment of newly diagnosed PML/RAR alfa-positive APL patients.

In the long-term follow-up, some sequelae have been described. The 10-year cumulative incidence of deaths in CR, resulting mainly from myelosuppression, has supported the need for less intensive myelosuppressive treatments, particularly for consolidation therapy (7).

Late complications after chemotherapy included therapy-related malignancies as therapy-related acute myeloid leukemia (t-AML) and myelodysplastic syndrome (t-MDS). t-MDS and t-AML patients usually showed poor responses to conventional chemotherapy with overall median survivals of 10 months (range 7–22 months) (8).

Arsenic trioxide (ATO), very effective as a single agent, was initially used for the treatment of the relapsed patients after treatment with ATRA and chemotherapy (9, 10). ATO has a different toxicity profile than ATRA and chemotherapy, but experimental studies tried to incorporate ATO into initial therapy and to reduce or omit the use of chemotherapy in selected patients.

Analysis of the cooperative group showed 3-year Kaplan-Meier curves with the estimation of relapse-free survival (RFS) (1). The resulting predictive model for RFS showed the capacity of segregating patients into statistically different groups (p<0.0001): low-risk (white blood cells WBC count ≤ 10 x 10^9^/L, platelet count > 40 x 10^9^/L), intermediate-risk (WBC count ≤ 10 x 10^9^/L, platelets ≤ 40 x 10^9^/L), and high-risk (WBC count > 10 x 10^9^/L) groups.

ATRA plus ATO without chemotherapy was an attempt in low and intermediate-risk APL patients; for high-risk APL triple therapy with limited anthracycline or gemtuzumab ozogamicin during induction was the principal option.

The introduction of arsenic trioxide (ATO) tried to obviate complications of chemotherapeutic agents in low and intermediate-risk groups. The use of ATO and ATRA showed excellent results in terms of CR and long-term leukemia free survival (11, 12).

Our study aims to describe a monocentric cohort of patients with newly diagnosed APL effectively treated with ATO plus ATRA analyzing efficacy and tolerability of this association.

## Materials and Methods

Patients with newly APL were diagnosed on the basis of morphologic features and confirmed by genetic and molecular characteristics. Translocation t(15,17) was detected by conventional karyotyping or fluorescence *in situ* hybridization (FISH) (13), *PML-RARA* fusion gene by means of reverse-transcriptase–polymerase-chain-reaction (RT-PCR) assay (14, 15).

The study was retrospective monocentric and it included all consecutive patients affected by APL and treated with ATRA plus ATO; few cases were treated also with idarubicin only in the induction phase.

According to Sanz’s risk score (1) they were classified as low, intermediate or high-risk.

Patients receive ATRA plus ATO for induction and consolidation therapy or standard ATRA–idarubicin induction therapy followed by ATRA-ATO for consolidation.

Data on patients who were still alive and in first molecular CR were censored at the most recent follow-up visit. Overall survival and cumulative incidence of relapse were defined according to the NCI workshop definitions (16).

Guidelines for the prevention and management of coagulopathy, hyperleukocytosis, prolongation of the corrected QT (QTc) interval (17), and hematologic and non-hematologic toxic effects were predefined in the protocols. As prophylaxis for the differentiation syndrome (18), prednisone at a dose of 0.5 mg per kilogram of body weight per day was administered from day 1 until the end of induction therapy. For suspected differentiation syndrome, ATRA, ATO or both were temporarily discontinued and intravenous dexamethasone was administered at a dose of 10 mg every 12 hours until the disappearance of signs and symptoms for a minimum of 3 days. Common terminology criteria for adverse events (CTCAE) version 4.0 was used for toxicity assessment.

## Results

From January 2009 to December 2019 we diagnosed 23 APL patients who were treated with ATO plus ATRA regimen: 14 males and 9 females patients with a median age of 45 years (range 18-72), for the majority intermediate risk (15 patients, 65%) but 4 patients were low-risk and 4 patients high-risk. Patients in the high-risk category according to Sanz’s criteria were treated with ATRA plus idarubicin (3 patients) and ATRA plus ATO with the addition of two doses of idarubicin (1 patient) in the induction. All the 23 patients were treated according to the regimen of ATO plus ATRA in the consolidation phase, included the 4 patients at high-risk, due to severe concomitant comorbidities.

The treatment of ATRA (started at the suspicion of APL) and ATO (started after a median of 2 days, range 1-5) was well tolerated: brief interruptions of ATO (≤ 3 days) were registered in 11 patients (in 7 for QTc prolongation, 3 for malaise, 1 for hepatic toxicity of grade 2). A possible retinoic acid syndrome was described in 10 cases; even if in many cases it was only suspected ATRA was discontinued for a brief period, patients were treated with steroids and then ATRA was restarted without any complications.

During the induction phase, we also registered 8 episodes of fever, 3 mild hepatic toxicities of grade 1 or 2, 2 thrombotic events (1 deep vein thrombosis and 1 thrombophlebitis of the leg), 2 viral infections sustained by HSV, 1 ATRA myopathy of grade 2 and 1 skin rash of grade 1.

All patients achieved molecular remission after a median time of 3 months (range 1-6 months).

All patients followed consolidation treatment as outpatients; only 4 patients were hospitalized: 1 patient was admitted for the suspicion of benign intracranial hypertension, 1 patient for pneumonia before starting consolidation, 1 patient for fever and pericardial effusion and 1 patient performed cholecystectomy as inpatient in surgical ward. During consolidation only 3 patients (13%) reduced ATO dosage for QTc prolongation; in two patients we registered 2 different episodes of QTc prolongation.

Other adverse events were mild hepatotoxicity (5 patients: 3 of grade 2 and 2 of grade 1), infections (cystitis in 2 patients, FUO in 2 patients, herpes in 1 patient, diarrhea grade 1 in 1 patient), pitting edema with fluid retention (3 patients) and headache (2 patients). All these adverse events were easily managed on outpatient ward; there was no delay in the treatment.

All patients maintained molecular response for a median time of 44 months (range 15-127) except for 1 patient who relapsed after 11 months: he restarted ATO plus ATRA achieving a second but brief molecular response and central nervous system localization; stem cell transplantation was carried out but the patient died from transplant related complications. All but one patient are alive and in response at a median follow-up of 48 months (range 9-141) without late effects.

## Discussion

ATO plus ATRA association shows advantages in comparison to chemotherapy: patients were hospitalized only during the induction phase and the rate of hospitalization and complications was very low and highly manageable. This regimen also allowed to treat high-risk patients in which chemotherapy could not be applicable such as secondary APL (due to prior chemotherapy or immunosuppressive agents), intracerebral hemorrhage or high rate of hemorrhagic symptoms and massive pulmonary embolism (19).

In the management of APL, it should be considered also the incidence of t-MDS and t-AML, which was considerable after a high-dose chemotherapy for a prior cancer and low in the case of patients treated by conventional therapy for APL, as only few cases have been described in literature. Some studies hypothesized the role of alkylating agents and etoposide to induce t-MDS or t-AML, but it was also investigated the leukemic potential of other drugs as anthracyclines. The median period to diagnose t-MDS or t-AML was 34 months (range 25-40) and all patients presented chromosome abnormalities (mainly deletions or loss of the long arm of chromosome 5 and/or 7, or balanced translocations involving the 21q22 band) and poor prognosis with median survivals of 10 months (range 7-22) (8).

We already published a report (20) in which we compared ATO plus ATRA with a control cohort treated with chemotherapy plus ATRA according to AIDA-2000 (12). Data on the historical group showed median molecular response time of 85 months (range 16-296) but 3 patients developed t-MDS/AML after 3 years, 6 years and 23 years from the end of the maintenance phase respectively, and 1 patient showed myelodysplastic features at bone marrow cytogenetics such as del5 after 12 years from the end of the maintenance phase. Two patients with t-MDS died for progressive disease.

Considering our comparison, the approach with ATO plus ATRA allowed to reduce hospitalization, transfusion support, early complications mainly related to consolidation phase and late complications such as t-MDS/AML whose progression could be fatal.

Exciting results are coming from clinical trials testing oral arsenic formulation (21) and they seem to further extend the benefit from ATO plus ATRA combination retaining excellent activity with the simple oral combination of these powerful agents and more clinical trials are ongoing to validate them.

Studies on health-related quality-of-life outcomes demonstrated for this regimen a better profile in terms of fatigue (22), with APL patients safely back to their normal daily living.

The association of ATO to ATRA as chemo-free regimen enabled to treat acute leukemia even without chemotherapy.

## Data Availability Statement

The raw data supporting the conclusions of this article will be made available by the authors, without undue reservation.

## Ethics Statement

Written informed consent was obtained from the individual(s) for the publication of any potentially identifiable images or data included in this article.

## Author Contributions

FA and SS designed the study, recorded clinical and biological data and wrote the manuscript. PC, FS, SG, LL, II, EM, NP, and LP contributed to the study design, recorded clinical and biological data and wrote the manuscript. All authors contributed to the article and approved the submitted version.

## Conflict of Interest

The authors declare that the research was conducted in the absence of any commercial or financial relationships that could be construed as a potential conflict of interest.
